# Effectiveness of the kiva antibullying program with and without the online game in chile: A three-arm cluster randomised controlled trial

**DOI:** 10.1192/j.eurpsy.2021.611

**Published:** 2021-08-13

**Authors:** J. Gaete, M. Valenzuela, S. Gana, C. Rojas-Barahona

**Affiliations:** 1 Faculty Of Education, Universidad de los Andes, Santiago, Chile; 2 Department Of Psychology Invest Research Flagship, University of Turku, Turku, Finland; 3 Faculty Of Education, Universidad de Talca, Talca, Chile

**Keywords:** effectiveness, randomized controlled trial, bullying, adolescents

## Abstract

**Introduction:**

Bullying is a major problem worldwide and Chile is no exception. Whole school-based antibullying programs offer an opportunity for preventing bullying at school. The KiVa antibullying program has been evaluated in Finland and other European countries, showing preventive effects on self-reported bullying victimization and bullying perpetration.

**Objectives:**

To test the effectiveness of a culturally adapted version of the KiVa antibullying program in socio-economically vulnerable schools in Santiago, Chile.

**Methods:**

We did a cluster randomized controlled trial in 5th and 6th graders at socially vulnerable schools. Schools were randomly assigned (1:1:1) to three groups: full KiVa group (including the online game), partial KiVa group (did not include the online game), and control group in which the regular school curriculum was implemented. The primary outcome was self-reported bullying victimization, measured with the Olweus Bully/Victim Questionnaire-Revised version (OBVQ-R). Students were assessed at the end of the academic year (November 2016) and 12 months later at the end of the academic year (November 2017). This trial is registered with ClinicalTrials.gov, number NCT02898324.

**Results:**

We included 39 schools (13 in each group). The baseline survey included a total of 5923 participants, and the endpoint survey included 3968 participants. Participants in the partial KiVa group had lower victimization and lower witnessing bullying at school at the endpoint survey than those in the control group. There was no effect of the full KiVa group. No effects were found for bullying perpetration in any of the comparisons between arms.

**Conclusions:**

The KiVa antibullying Program had small effects in its implementation in Chile.

